# Hydrogeochemical processes and water quality of the Aïn Taga Karst Spring, Tlemcen, Northwestern Algeria

**DOI:** 10.1007/s10653-026-03286-6

**Published:** 2026-06-16

**Authors:** Sabrine Guettaia, Abderrezzak Boudjema, Abdessamed Derdour, Abdessalam Laoufi, Ashraf A. Ahmed, Abdulrahman Seraj Almalki, Ahmed A. Arafat, Sherif S. M. Ghoneim

**Affiliations:** 1 Laboratory N°25 Promotion of Water, Mineral and Soil Resources, Environmental Legislation and Technological Choices, University of Tlemcen, P.O. Box 119, 13000 Tlemcen, Algeria; 2 Artificial Intelligence Laboratory for Mechanical and Civil Structures, and Soil, University Center of Naama, P.O. Box 66, 45000 Naama, Algeria; 3 Department of Civil and Environmental Engineering, Brunel University of London, Kingston Lane, Uxbridge, UB83PH UK; 4 Civil and Environmental Engineering Department, College of Engineering and Computing in Al-Qunfudhah, Umm Al-Qura University, Mecca, Saudi Arabia; 5 Department of Civil Engineering, College of Engineering, Taif University, P.O. Box 11099, 21944 Taif, Saudi Arabia; 6 Department of Electrical Engineering, College of Engineering, Taif University, 21944 Taif, Saudi Arabia

**Keywords:** Aïn Taga Spring, Tlemcen, Hydrogeochemical, Karst system, Vulnerability, Anthropogenic pollution

## Abstract

This study presents the first long-term hydrogeochemical monitoring (2014–2024) of the Aïn Taga karst spring, located in the Ghar Boumaâza karst system (northwestern Algeria). The analysis is based on 71 samples, examined for major ions, salinity, hardness and saturation indices, interpreted using Piper and Chadha diagrams, multivariate statistical methods (PCA, CAH) and mineral balance calculations. The results reveal a clear dominance of the Ca–Mg–HCO₃ facies (94.4% of the samples), indicating that carbonate dissolution mainly controls mineralization, with waters supersaturated with calcite and dolomite and a limited influence of evaporitic formations. A cubic polynomial model HCO₃⁻—Q highlights a nonlinear relationship between bicarbonate concentration and flow, characterized by high concentrations at low flow rates (prolonged residence times and strong water–rock interaction) and progressive dilution during high-water episodes, with very good predictive performance (R^2^ ≈ 0.95, MAE ≈ 15 mg/L, RMSE ≈ 19 mg/L). Principal component analysis clearly distinguishes natural mineralization from low anthropogenic inputs (NO₃⁻, Cl⁻, SO₄^2^⁻), while the marked negative correlation between flow and bicarbonate (ρ =  − 0.66) highlights the dilution processes during floods. In this strategic, highly vulnerable and historically understudied karst, this integrated approach provides an operational framework to link the hydrodynamic regime and hydrochemical signature, improve the interpretation of carbonate dissolution processes, identify localized contaminations and guide monitoring and protection strategies for water resources.

## Introduction

Karst areas, which cover only a small fraction of Earth's surface (7–12%), provide nearly a quarter of the world's drinking water (Ford & Williams, 2007; Goldscheider et al., 2007). Karst aquifers form from the dissolution of lithologic units and can develop complex fissure and conduit systems, making them highly permeable and capable of storing large amounts of water (Hartmann et al., 2014; Jodar-Abellan et al., 2024).

However, this strong heterogeneity, combined with high permeability, makes these systems highly vulnerable to surface contamination and limits their self-cleaning capabilities (Nanou & Zagana, 2018). As a result, the chemical structure of waters in these karst environments is determined by a set of natural geochemical interactions: lithology, hydrometeorological input, subsurface circulation, and the growing impact of anthropogenic processes (Leins et al., 2025). To better understand the development and vulnerability of these systems, the mechanisms of chemical elements, particularly bicarbonates as a representative of dissolved matter fluxes, need to be studied, since these processes directly influence karst weathering and water quality (Klimchouk et al., 2017). It has also been widely reported that turbidity, particle transport, and mineral saturation are the main drivers of this phenomenon (Hartmann et al., 2014; Li et al., 2025).

Other factors, such as temperature, pH, and the presence of salty water, also affect carbonate dissolution, thereby changing porosity and structural stability in karst environments (Dreybrodt, 2017; Massei et al., 2017). All of these interactions define the correlation between the total dissolved solids (TDS) concentration and groundwater discharge (Q) nexus, which is carefully studied to track weathering in the aquifer and hydrodynamic responsiveness (Goldscheider et al., 2007; Guettaia et al., 2025).

The specific determination of the rate of bicarbonate evolution with discharge is essential to understanding transfer processes and changes in karst systems, especially the role of carbonate dissolution (calcite and dolomite) in mineralizing water (Zerga, 2024). It is possible to identify time periods or situations in which dissolution processes dominate, distinguish between natural and human inputs, and understand the dynamics of chemical weathering in karst landscapes by examining the variation in bicarbonate concentration with flow (Hanshaw & Back, 1979). Such an approach enables predicting changes in water quality based on hydraulic variations, approximating the system's stability under climate change or anthropogenic pressures, and informing water resource management and protection plans in endangered areas, such as Ghar Boumaâza (Guettaia et al., 2025).

It therefore follows that dissolved bicarbonate loads are significant for estimating changes in the discharge ratio and for rational governance of water resources. However, traditional sampling and chemical analysis methods are often costly, time-consuming, and spatially limited (Bouarfa et al., 2022; Derdour et al., 2017). To overcome these limitations, modern studies use hydrogeochemical techniques combined with multivariate statistical methods, which enable more precise predictions of dissolved matter concentrations and the explanation of the processes that govern water quality.

These techniques are also useful for identifying key threats, pollution, and ecological and in establishing vulnerability gradients in karst environments. The VUKA method is one of the tools that illustrate the effectiveness of these methods in defining the vulnerability of North African karst aquifers to contamination (Leyland & Witthüser, 2010). Additional multivariate statistical tools, such as the principal component analysis (PCA), are also used to help separate natural and anthropogenic sources of dissolved elements (Moussaoui et al., 2024). Regression equations between bicarbonate concentration (HCO3^−^) and discharge (Q) have been derived as a salient measure for assessing the aquifer's response to hydrologic processes and dissolution (Yidana et al., 2012).

The study integrates multivariate statistical analyses (correlations, PCAs, predictive modeling) to reveal the links between the different chemical parameters, to distinguish between natural and anthropogenic contributions, and to model the evolution of karst weathering via the relationship of bicarbonate concentration as a function of hydrological variables (such as flow). This approach provides a transferable methodological framework for assessing and sustainably managing vulnerable karst aquifers, especially in semi-arid environments.

Located in the Tlemcen Mountains, the Ghar Boumaâza karst network—the largest known underground system in Algeria, with more than 4 km of development—now presents a combination of marked pressures, between overexploitation of the resource, overgrazing and diffuse pollution. In contrast, more than 66% of its surface area is classified as a high vulnerability zone. Despite its strategic importance, this karst has been the subject of very few studies since the 1980s, except the more recent work of Bensaoula et al. (2017) and Guettaia et al. (2025).

The present study stands out by relying on an unprecedented multi-year hydrochemical dataset (71 samples collected between 2014 and 2024 at the Aïn Taga Spring, the main resurgence of Ghar Boumaâza) to characterize in an integrated way the hydrochemical processes that control water quality and the internal functioning of the system. It proposes an original methodological framework combining, on the one hand, a nonlinear flow–chemistry model based on a cubic regression HCO₃⁻–Q, capable of reproducing both strong mineralization at low flow rates (extended residence times in the reservoir) and progressive dilution during flood periods with high statistical performance, and on the other hand, the systematic application of multivariate statistical tools (Pearson/Spearman correlations, ascending hierarchical classification, principal component analysis).

This combination allows, for the first time on this karst system, to quantitatively discriminate the natural contributions related to the dissolution of carbonates (Ca–Mg–HCO₃⁻ facies, supersaturation with respect to calcite and dolomite) from the anthropogenic point inputs (NO₃⁻, Cl⁻, SO₄^2^⁻, Na, K) associated with agricultural practices and domestic discharges. The approach developed thus provides a transferable framework for simultaneously diagnosing the mixture of diffuse and rapid flows, residence times, mineral saturation state and degree of vulnerability to surface pressures, offering a significant advance compared to previous studies on the Ghar Boumaâza area and an operational tool for the sustainable management of karst aquifers in a semi-arid environment.

## Materials and methods

### Description of the study area

**Fig. 1:**
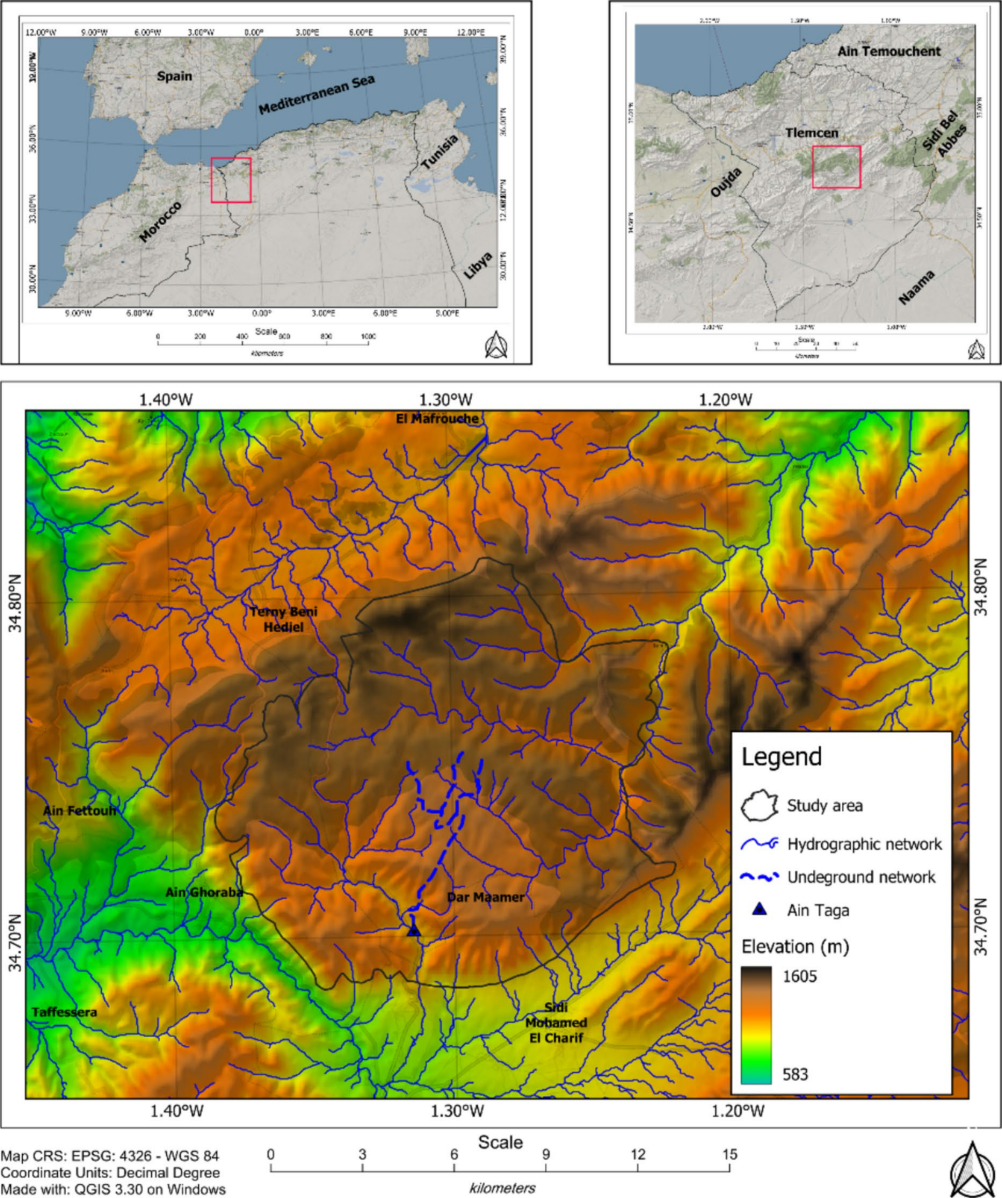
Location map of the Ghar Boumaâza karst system in the Tlemcen Mountains, Algeria

Located in the Tlemcen Mountains, near the town of Sebdou, the Ghar Boumaâza karst system is located at the following geographical coordinates: latitude 34°37' N and longitude 1°20' W, about 20 km from the city of Tlemcen, the main urban center of the region (Fig. 1). The population of Tlemcen region has increased significantly over recent decades, which has intensified strain on the amount of water particularly the Ghar Boumaâza karst system (Guettaia et al., 2025). Water supply in the area relies on several boreholes, mainly used for drinking water, as well as karst springs, among which Aïn Taga and Aïn Hassi El Kelb are the most important. However, these water resources are highly vulnerable to anthropogenic pollution, mainly of domestic origin, as reported in the area of Dar Maâmar (Bensaoula, 2008). This case shows the vulnerability of karst aquifers to human activities related to pollution, which is why stringent, vulnerability-adjusted management is necessary.

### Geological and hydrogeological setting

**Fig. 2:**
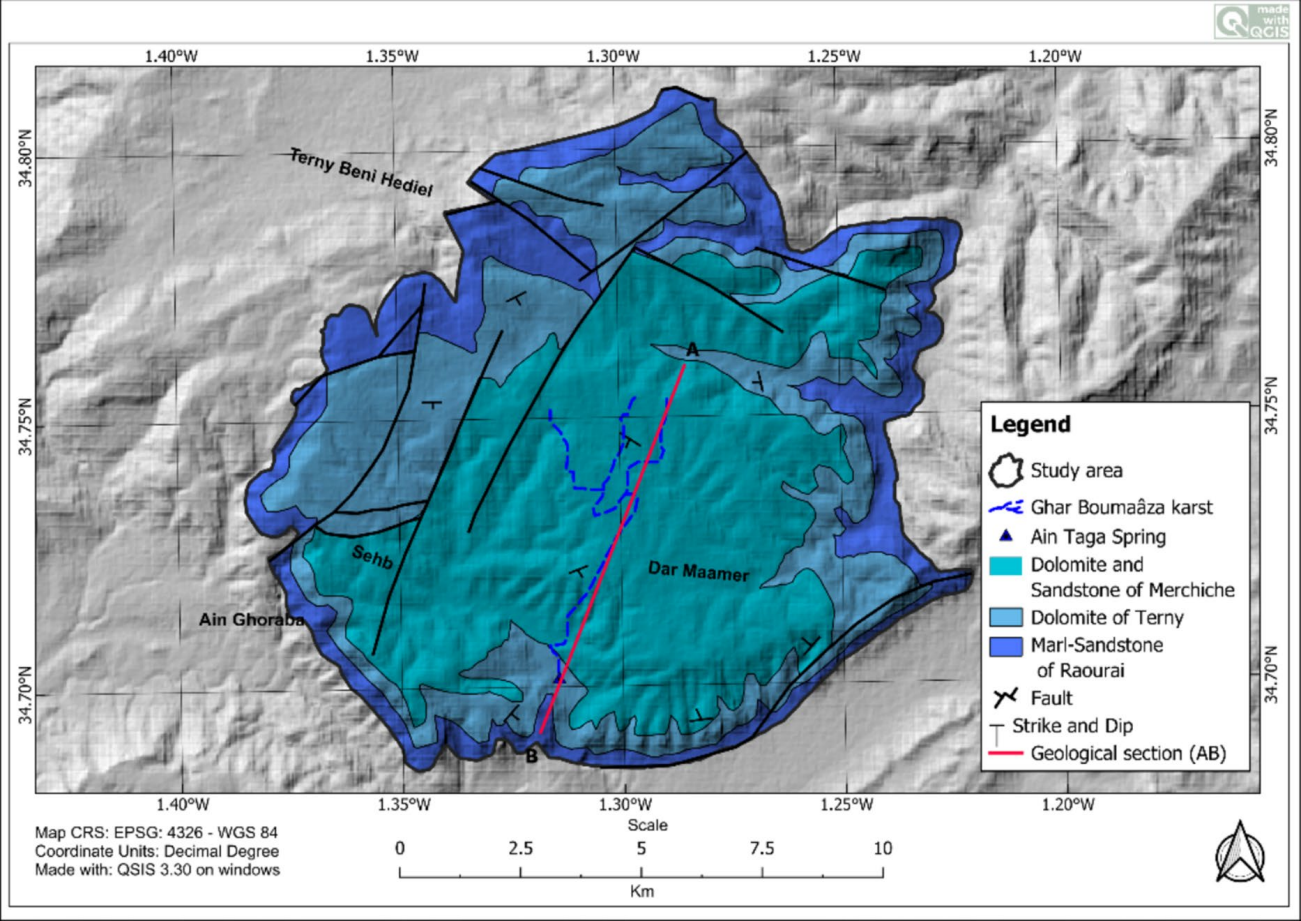
Geological map of the Ghar Boumaâza region (Guettaia et al., 2025)

**Fig. 3:**
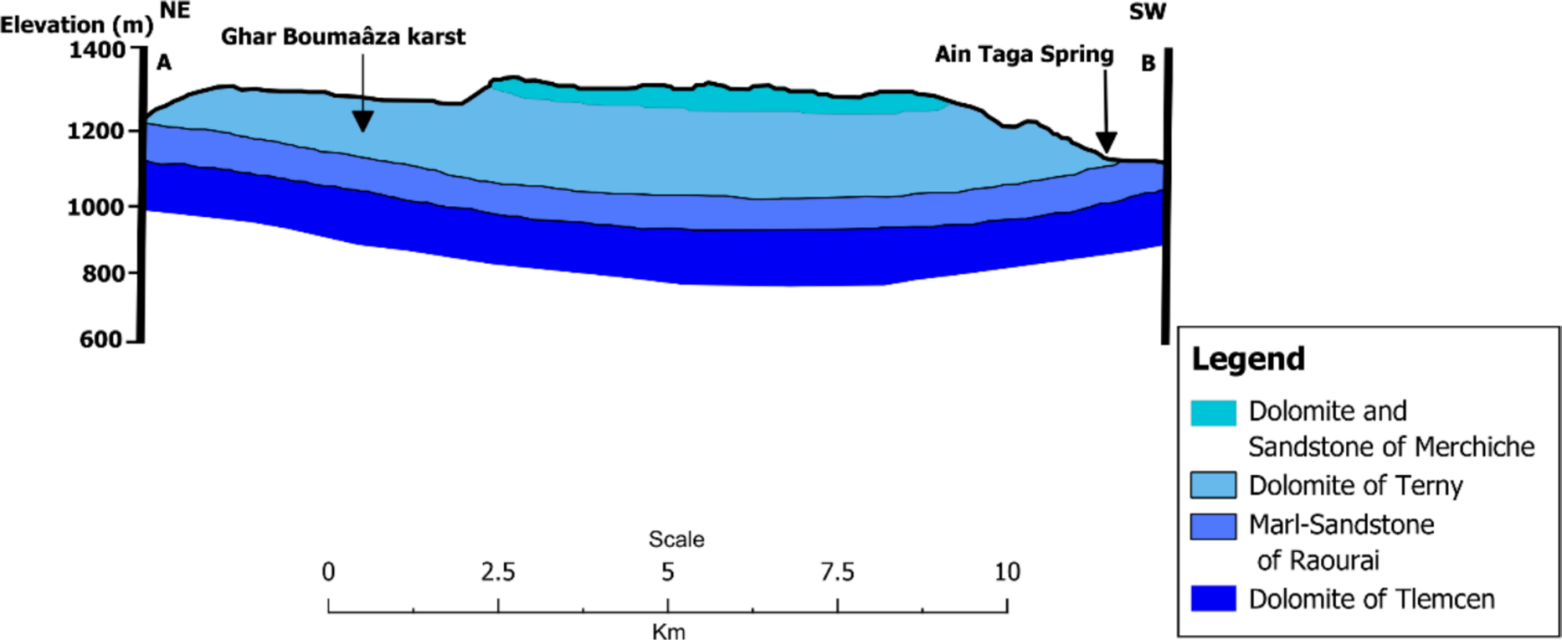
Cross-section AB through the Merchiche syncline

The Ghar Boumaâza karst system has developed within calcareous-dolomitic formations (Fig. 2), structured in a southwest-northeast-oriented syncline known as the Merchiche syncline (Fig. 3). According to Benest (1984), these geological formations follow one another from the base to the summit in the following way: first the Terni dolomites (DTe, 100 m thick), then the Hariga marl-limestones (ZA), composed mainly of limestone over 195 m, topped by a sandstone level of 52 m corresponding to the Merchiche sandstones (Azzaz et al., 2008). These sandstone (ZB) layers contribute to the recharge of small springs with a moderate flow, which are used both for local consumption and for watering livestock (Mamoune et al., 2023). Altogether, these formations represent approximately 347 m of mainly calcareous deposits, which are often difficult to identify due to dolomitisation. They rest on an impermeable base made up of the Raourai marl-limestone (MC), which forms the bottom of the aquifer and in which the galleries of the Ghar Boumaâza network have been dug (Collignon, 1986). This complex geological structure also rests on the Tlemcen dolomites (Dl), which are locally karstified, and exemplifies a type of Mediterranean karst system shaped by an active geological evolution that controls its present‑day hydrological functioning (Laoufi et al., 2025).

The Ghar Boumaâza karst system is described as a perched aquifer, with the main exit points being the AïnTaga and Ain Hassi El Kelb springs, located about 500 m downstream of the temporary resurgence of this cave, which provides access to the underground river of the same name (Bensaoula, 2008). A tracing test conducted by Birebent in the 1950s, from a loss located 1600 m from the cave entrance, showed that water reappears at the Aïn Taga spring after only a few days (Collignon, 1986). According to long-term data collected at the Meffrouch station between 1976 and 2022, on the northern slope of the area, the Ghar Boumaâza site enjoys a temperate semi-arid Mediterranean climate, with notable continental influences (Guettaia et al., 2025). Flow measurements, regularly carried out by the National Agency for Hydraulic Resources (ANRH) at the network outlet, show particularly marked fluctuations (ranging from 0 to 4000 L per second) closely linked to rainfall patterns (Bensaoula et al., 2017). This variability illustrates the karst system’s strong reactivity to climatic variations. Physico-chemical analyses indicate that the water in this karst is predominantly calcium bicarbonate, with low mineralization (300 mg/l) (Bensaoula et al., 2017). While the overall physico-chemical quality of groundwater remains satisfactory, the situation is more worrying regarding bacteriological quality, particularly in the Dar Maâmar area (Guettaia et al., 2025).

### Sampling and testing

This research is based on a dataset of seventy-one groundwater samples collected from the Aïn Taga spring. This permanently supplied spring is the main outlet point for the waters of the underground network of Ghar Boumaâza, a system of caves and galleries dug into the limestone rock. All samples were collected at the spring outlet (Aïn Taga) to document natural variations in water supply. Sampling took place over 10 years, from January 2014 to December 2024, except during the pandemic period. This long duration allows for the seasonal and year-to-year variations. Under normal conditions, sampling was performed at intervals of approximately 1.5–2 months, during dry or rainy periods. During significant hydrological events, the frequency increased to one sampling every two weeks. This strategy allows us to understand how this type of underground source works.

The spring in Aïn Taga shows significant changes in discharge, a characteristic trait of karstic aquifers fed by calcareous substrates in a semi-arid climate. With antecedent precipitation, discharge can increase to more than 2000 l/s, but during dry conditions may drop to as low as 1.5 l/s. These variations can be especially explained by the lithological characteristics of the limestone subsoil, which responds quickly to rainfall, mobilizing water into preferential flow toward the spring. Hydrological data were provided by the National Agency for hydraulic Resources (ANRH) of the Tlemcen region of Algeria. Situation measurements of the main characteristics of the water, total dissolved solids, electrical conductivity, and pH, were taken. The study was focused on eight key chemical elements naturally present in the water: four cationic (Na^+^, K^+^, Ca^2+^, Mg^2+^) and four anionic (HCO_3_^−^, Cl^−^, SO_4_^2−^, NO_3_^−^) species. Certified hydrogeologists of the National Agency, following strict quality-control measures, oversaw the sampling, preservation, and transportation of the samples. All chemical analyses determinations were conducted in the agency's official laboratories, the only institution permitted by Algerian law to assess the quality of groundwater for agricultural use.

After ion concentrations were determined, analytical errors (AEs) were calculated using Eq. (1).

1 $$IB(\% ) = \frac{{\left[ {\sum {cations - \sum {anions} } } \right]}}{{\sum {cations + \sum {anions} } }}x100$$

where:

- Cations milliequivalents per liter (meq/L) include positively charged ions, e.g., calcium, magnesium, sodium, and potassium, which add to the net positive charge of the solution.
- Anions, also given in milliequivalents per liter (meq/L), are negatively charged ions, such as bicarbonate, carbonate, chloride, sulphate, and nitrate, which neutralise the total ionic charge of the sample.
- The acceptable ion balances normally have a deviation within a range of less than ± 5% of good analytical results. Thresholds above this level may indicate sampling, analysis, or calculation errors (Nordstrom et al., 1989).

### Chart analysis

Hydrochemical diagrams, such as Piper and Chadha plots, and the ascending hierarchical classification (AHC), are essential tools in hydrogeology, especially for evaluating karst systems (Boudjema et al., 2024). These approaches can easily convert complex analytical outputs into clear visualizations, making it easier to comprehend mineralisation processes, the geochemical origin of waters, hydrologic-geologic interactions, and the temporal and spatial dynamics of recharge and aquifer development (Guettaia et al., 2025).

An example of this is the Piper diagram, which consists of two ternary triangles (one representing cations, the other representing anions) connected by a central diamond (Piper, 1944). His schematic allows one to create a general portrait of the water chemistry and keep track of the hydrochemical facies changes in space and time. On the other hand, the Chadha diagram provides a more insightful interpretation of water types and allows for the depiction of major hydrochemical processes. In line with this, it can be used to determine groundwater chemistry determinants and indicate spatial or temporal changes.

Ascending hierarchical classification is a statistical approach that groups samples based on their chemical similarity, which results in a dendrogram (Ward, 1963). This method allows differentiation of discrete hydrogeochemical families, emphasises shared mineralising processes, and makes the analysis of a complex dataset more readable, facilitating inter-group communication and distinctions.

### Statistical analyses

Graphical analyses can detect sample groups with similar chemical signatures, while a statistical study based on descriptive analyses, correlation matrices, specific bivariate analyses (Pearson and Spearman coefficients, root mean square error), multivariate analysis, and regression modeling aims to explain the relationships between key parameters and confirm relevant patterns (Boudjema et al., 2024). The dataset are first subjected to descriptive statistics (mean, median, standard deviation, minimum, maximum) to characterize the distribution and variability of each hydrochemical variable. A general correlation table is then established for all quantitative variables, using Pearson's coefficients for linear relationships and Spearman's coefficients for monotonic relationships, with statistically significant correlations summarized in a dedicated table. Five major pairs of geochemical variables are examined in detail: total hardness–Ca^2^⁺, salinity index–SO₄^2^⁻, mineralization potential–Ca^2^⁺, Ca^2^⁺–HCO₃⁻, and Mg^2^⁺–HCO₃⁻, for which correlation coefficients, significance tests, and error indicators are calculated.

**Fig. 4:**
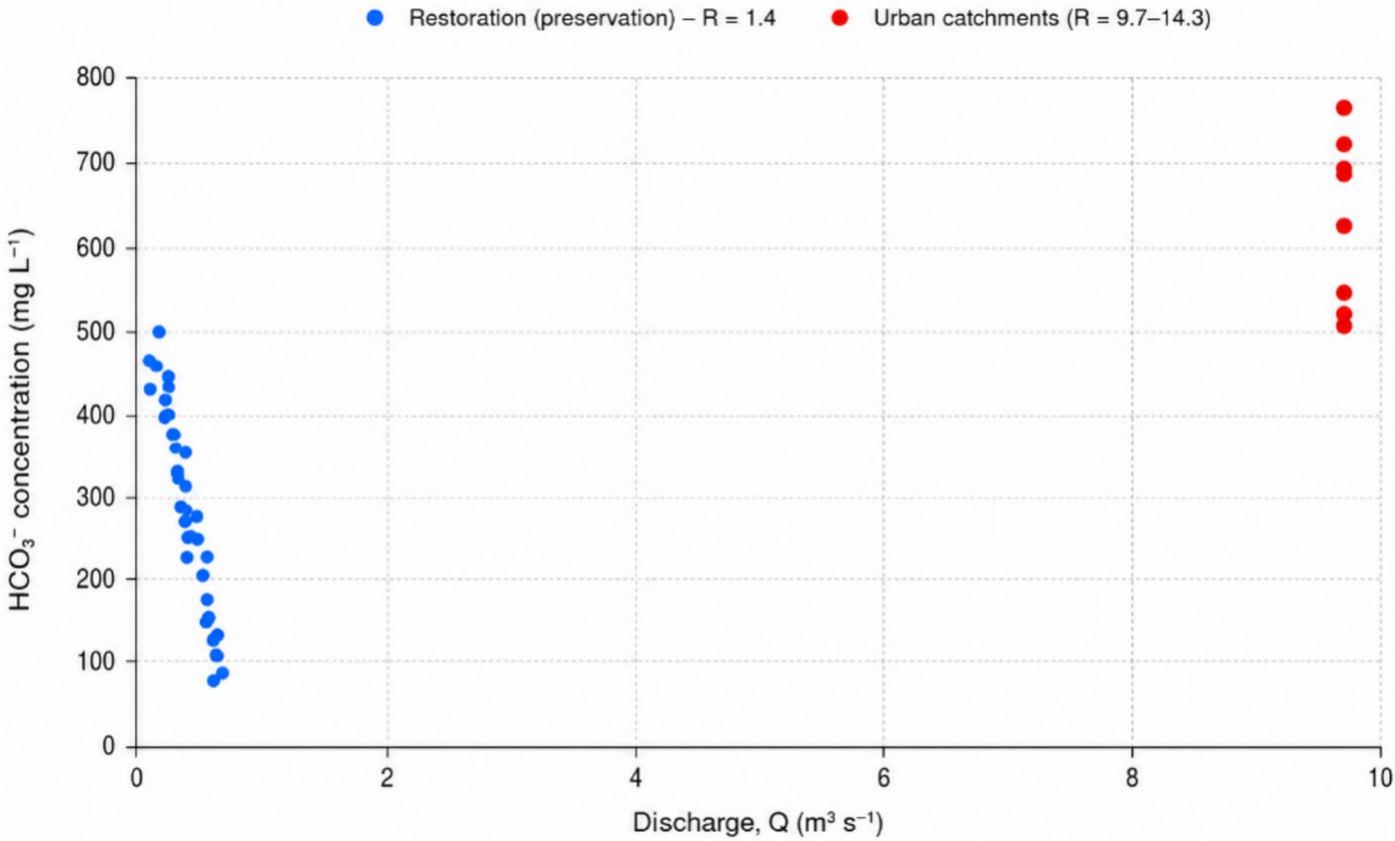
Relationship Between Discharge and Bicarbonate concentrations

In the initial dataset, some observations show very high flow rates (Q ≈ 9.7 L/s) associated with HCO₃⁻ concentrations above 350 mg/L, which deviate from the main point cloud, which is concentrated for Q < 1 L/s (Fig. 4). These values are both outside the interquartile range of flow rates and at the end of the HCO₃⁻ range, making them highly influential for a polynomial fit of degree 3. From a statistical point of view, these observations have a significant leverage effect and can constrain the curvature of the polynomial artificially, by degrading the fit on the most frequent operating area of the system (Q < 1 L/s). In hydrological practice, these situations correspond to extreme episodes (floods, transient mixing conditions, or measurement or input errors) that do not reflect the basic regime sought for the mean HCO₃⁻–Q relationship. The elimination (or, at the very least, the specific treatment) of these outliers is therefore justified by:

1. The need to preserve the representativeness of the model in the domain where the majority of the observations are located,
2. The reduction of the disproportionate influence of a few extreme points on the polynomial coefficients,
3. Consistency with the hydrogeochemical objective: to describe the average behaviour of the system rather than exceptional episodes that are not very reproducible.

A third-degree regression is estimated to provide a nonlinear model of the dependence between HCO₃⁻ and flow (Q) (Chen et al., 2022).

The evaluation of the model is not based solely on the coefficient of determination (R^2^), but also includes error indicators such as root mean square error (RMSE) and mean absolute error (MAE), which directly quantify the mean deviation between observed and predicted values across the training and test sets. Despite the apparent quality of the polynomial fit, the use of a high-order polynomial remains essentially empirical and should not be overinterpreted in the absence of a clear physical justification; the model is used as a numerical approximation of the HCO₃⁻ vs Q relationship rather than as a mechanistic representation of the transfer and mixing processes (Boudjema et al., 2024).

From a physical perspective, the nonlinear response of HCO₃⁻ to flow can be explained by the dilution and mixing mechanisms between different flow components in the karst system. At low flows, drained water is dominated by slow contributions from epikarst and matrix zones, which are highly mineralized in HCO₃⁻ due to long residence times and favorable conditions for carbonate dissolution. During episodes of higher flow, rapid inflows of runoff or preferential infiltration water, which are weaker in minerals, mix with base waters and induce dilution. However, this dilution is not proportional to the flow, because the relative proportion of the different reservoirs (baseflow, interflow, quickflow) varies in a non-linear manner with the intensity and dynamics of the rainfall episodes. The result is a curved relationship between HCO₃⁻ and Q, marked by areas of saturation, thresholds, and transitions corresponding to distinct flow regimes rather than a simple linear dilution.

Finally, principal component analysis (PCA) is used to identify the main mineralization processes and distinguish between natural and anthropogenic influences, providing an overview of karst functioning and groundwater quality (Guan et al., 2025). This approach makes it possible to relate the variables from the bivariate and regression analyses to the major hydrochemical gradients (carbonate dissolution, cation exchange, mineral precipitation, anthropogenic inputs). It also enables the integration of quantitative results (correlations, error metrics, residue diagnostics) into a holistic interpretation of the processes controlling the chemical composition of the source.

**Fig. 5:**
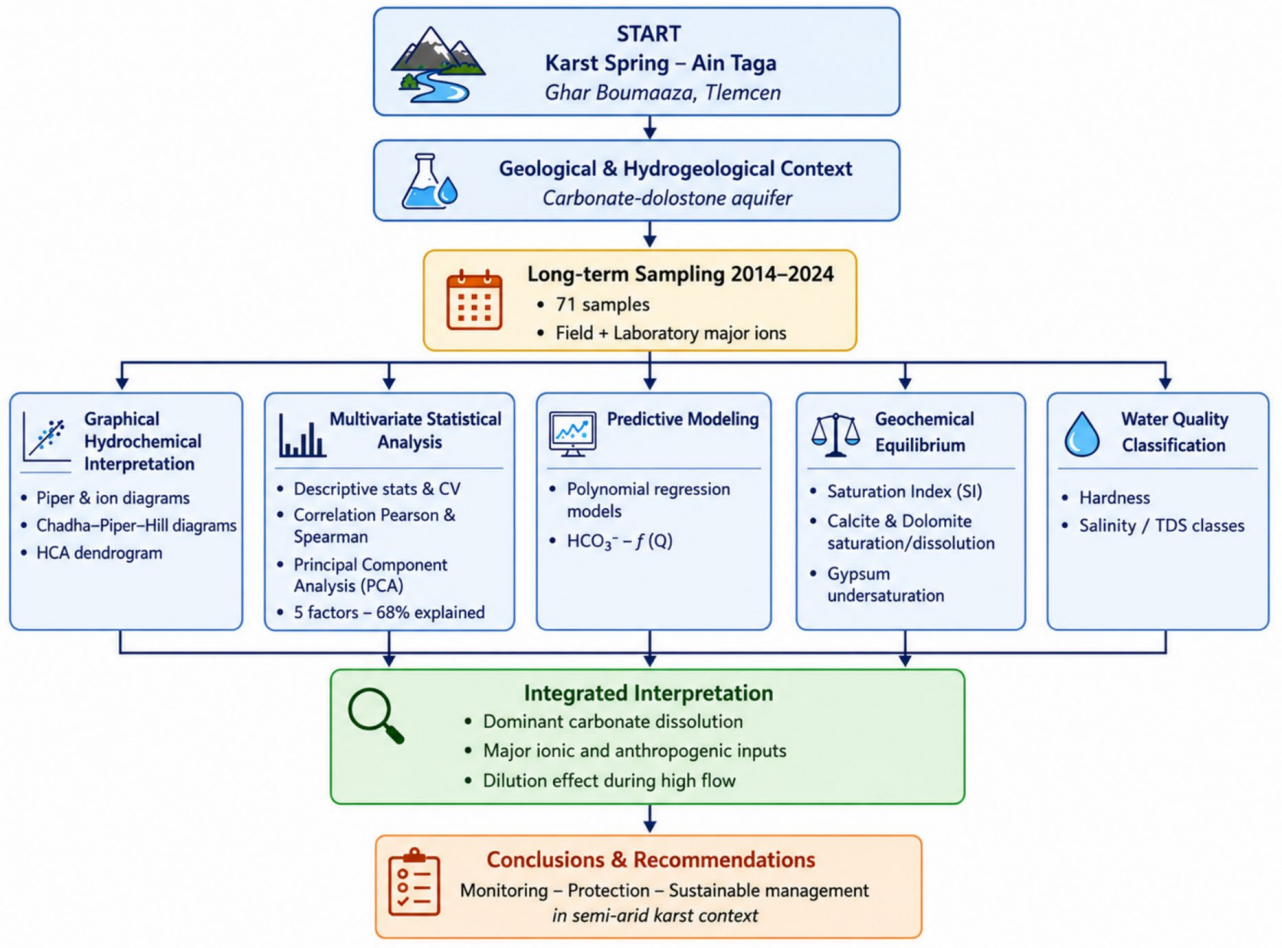
Schematic diagram of the proposed methodology

The research follows an integrated methodological framework designed to characterize the hydrogeochemical functioning and vulnerability of the Aïn Taga karst spring. As illustrated in Fig. 5, the approach begins with an analysis of the regional geological and hydrogeological context of the carbonate-dolomitic aquifer. This is followed by a long-term hydrochemical monitoring program (2014–2024), involving the collection and laboratory analysis of 71 water samples for major ions. The data processing stage employs a multi-pronged strategy: graphical interpretation using Piper and Chadha diagrams alongside HCA dendrograms; multivariate statistical analysis including descriptive statistics and Principal Component Analysis (PCA); and predictive modeling via a third-degree polynomial regression to define the HCO_3_^−^ vs. Q relationship. Finally, geochemical equilibrium is assessed through saturation indices for calcite, dolomite, and gypsum, leading to an integrated interpretation of dominant dissolution processes, anthropogenic impacts, and dilution effects.

## Results

### Graphical analysis

#### Piper diagram

**Fig. 6:**
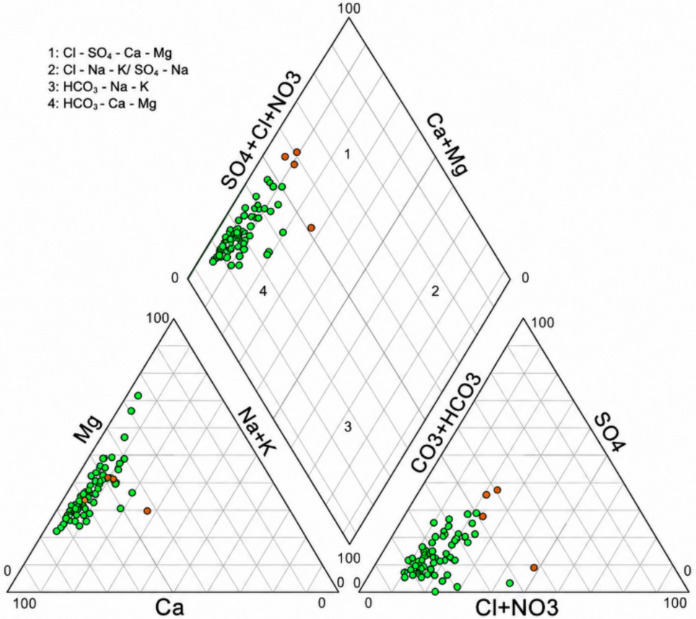
Piper diagram representing the ionic composition of groundwater samples from Aïn Taga Spring

The Piper (1944) diagram is a commonly used tool for categorizing and illustrating the chemical composition of water samples based on their predominant ionic concentrations (Fig. 6). The analysis results reported in this diagram indicate that the HCO_3_^−^–Ca^2+^–Mg^2+^ facies is predominant, accounting for 94.4% of the water samples. The groundwater in the study area contains mainly substantial amounts of calcium, magnesium, and bicarbonate ions. This facies is frequently associated with groundwater that has undergone significant interaction with carbonate rocks, leading to the decomposition of calcium and magnesium carbonates. This water usually means recharge zones, when rainfall seeps into the soil and solubilizes carbonate minerals. The Cl^−^–SO_4_^2−^–Ca^2+^–Mg^2+^ facies is represented by 5.6% of the samples, presented by samples 7, 13, 22, 33 indicating a significant impact of chlorides and sulphates, which may indicate that mineralization is due to the presence of evaporitic formations or anthropogenic pollution.

#### Chadha diagram

**Fig. 7:**
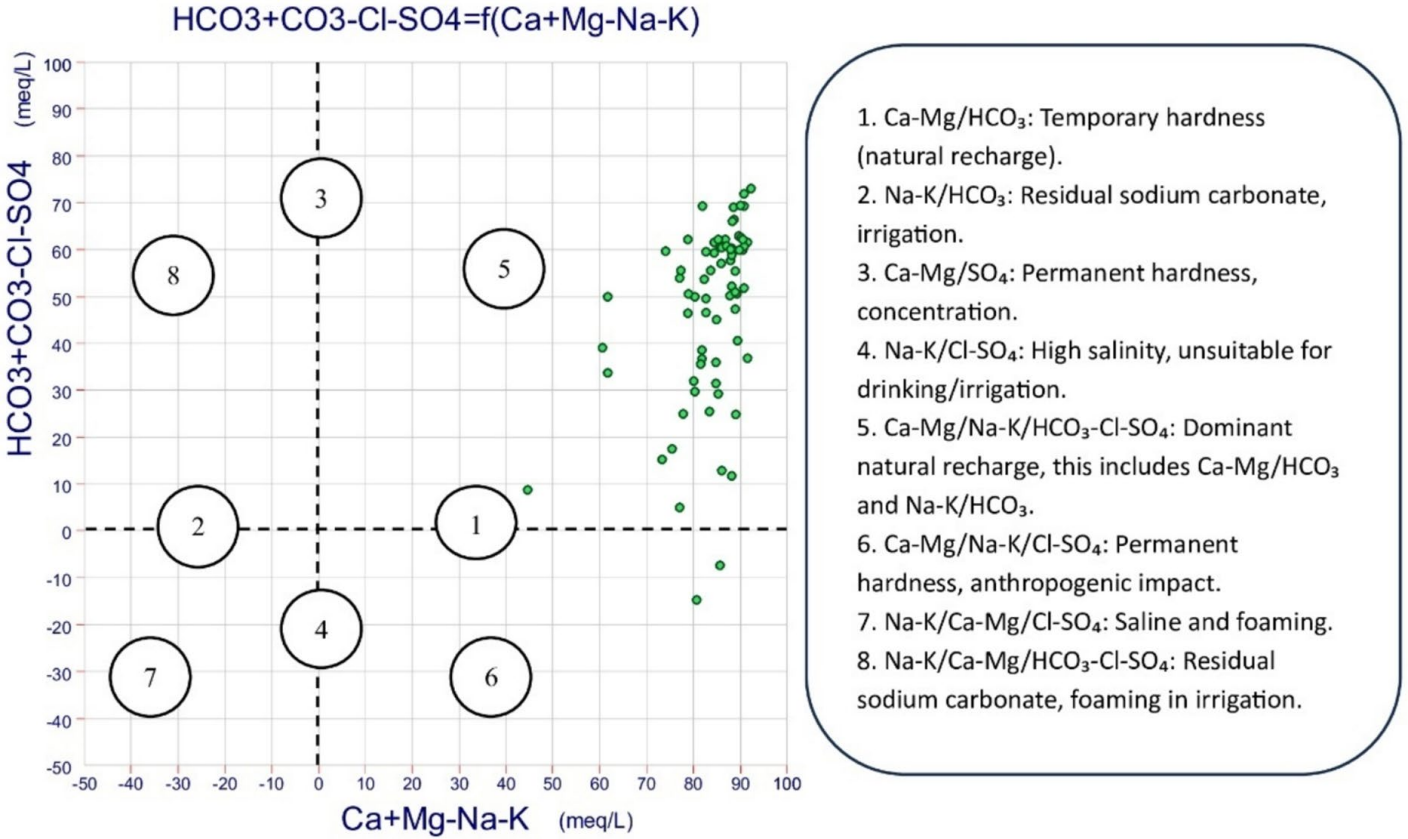
Chadha diagram for the classification of groundwater types and the identification of dominant hydrochemical processes

The hydrochemical diagram of Chadha (1999) classifies groundwater according to the relative proportions of the main cations (Ca^+^, Mg^+^, Na^+^, K^+^) and anions (HCO_3_^−^, Cl^−^, SO_4_^2−^), each field in the diagram corresponding to a hydrochemical facies illustrating either natural geochemical processes or anthropogenic influences (Fig. 7). The analyses show that almost all the samples are located in field 5 (Ca-Mg/Na–K/HCO_3_^−^ Cl^−^ SO_4_^2−^), which reflects a dominant natural recharge, associated with a hardness that is most often temporary. In contrast, only samples 13 and 33 belong to field 6 (Ca-Mg/Na–K/Cl^−^SO_4_), which is characterized by permanent hardness and human-induced impacts, often associated with increased sulphate and chloride contents. These results indicate that the chemical composition of water is mainly influenced by natural phenomena, such as the dissolution of minerals or the infiltration of meteoric water, thereby ensuring good-quality water suitable for consumption, irrigation, and industry without special treatment. However, the identification of potential anthropogenic contamination in these two samples highlights the need for enhanced monitoring, precise identification of pollution sources, and implementation of appropriate management and protection measures to sustainably preserve the groundwater resource while monitoring local changes in water quality.

#### Ascending hierarchical classification (CAH)

**Fig. 8:**
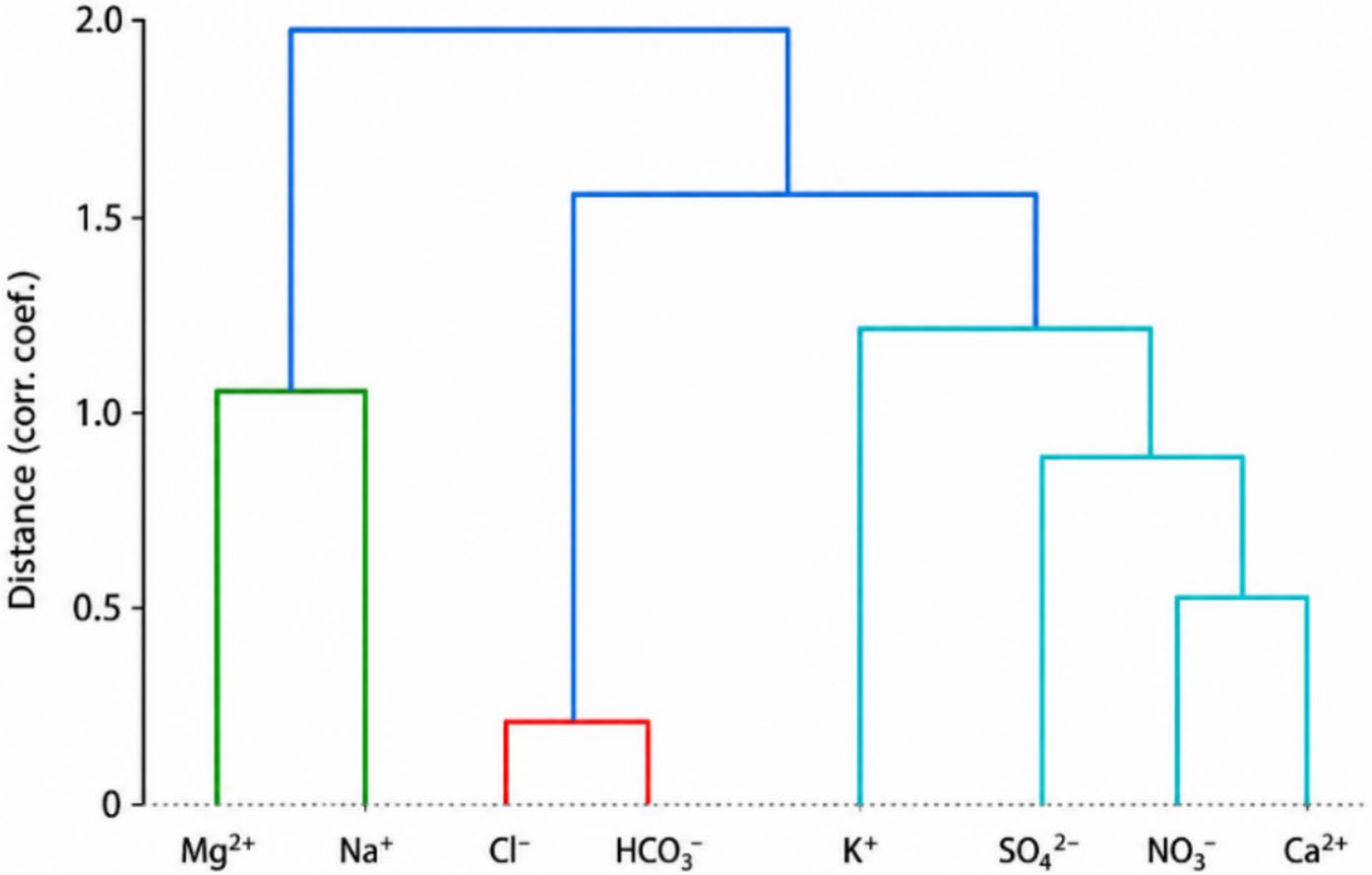
Ascending hierarchical classification (HAC) dendrogram of water samples

The analysis of the 71 water samples was carried out using the Ward Jr (1963) method with correlation distance to classify the chemical parameters based on their similar behaviour, allowing the identification of those that evolve in a coordinated manner. The dendrogram obtained (Fig. 8) reveals that the mineralization of the karst waters is dominated by the dissolution of limestone (Ca^2^ +–HCO_3_^−^), supplemented by inputs of dolomite or accessory minerals (Mg^2^–Na⁺) and strongly influenced by anthropogenic inputs (NO_3_⁻, SO_4_^2^⁻, K⁺, Cl^−^), mainly of agricultural and urban origin. These groupings reflect the diversity of geochemical processes and the vulnerability of karst aquifers to environmental pressures.

### Statistical analysis

#### Descriptive statistics and data quality

Descriptive statistical analysis of the chemical elements of the waters of the Aïn Taga spring reveals a temporal variability ranging from low to significant in their distribution (Table 1). A high coefficient of variation of more than 40% characterizes ions with high relative concentration variability, particularly sulphates (SO_4_^2−^), sodium (Na +), and chlorides (Cl⁻), suggesting the influence of localized contamination sources, variable ion-exchange processes, or differential mixing between distinct water bodies. Conversely, a moderate coefficient of variation between 20 and 40% indicates a lower and more homogeneous dispersion of concentrations, as observed for calcium (Ca^2^⁺), magnesium (Mg^2^⁺), and bicarbonates (HCO3-), revealing more stable and uniform geochemical processes related to the basal dissolution of carbonate minerals throughout the aquifer system.

**Table 1:** Descriptive statistical analysis of the major elements on the waters of the Aïn Taga spring

Variable	Min (mg/l)	Max (mg/l)	CV (%)	Variable	Min (mg/l)	Max (mg/l)	CV (%)
Ca^2+^	63.00	242.00	32.44	Cl⁻	0.23	82.00	44.66
Mg^2+^	0.27	39.00	24.15	SO4^2^⁻	1.00	127.00	59.60
Na^+^	5.00	35.00	54.25	HCO3⁻	120.00	507.56	32.00
K^+^	1.00	4.00	42.08	NO3⁻	7.00	55.00	40.95

#### Global correlation matrix

**Fig. 9:**
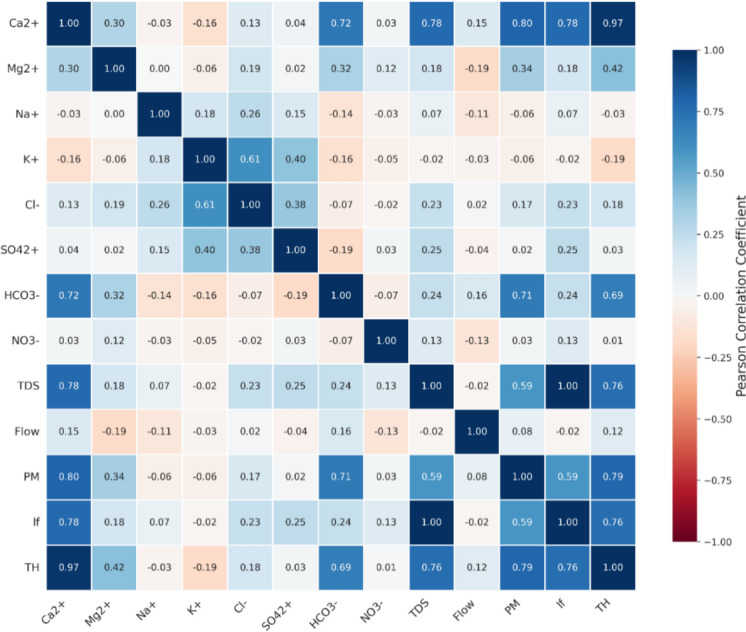
Pearson correlation matrix between the main physicochemical parameters of groundwater

**Fig. 10:**
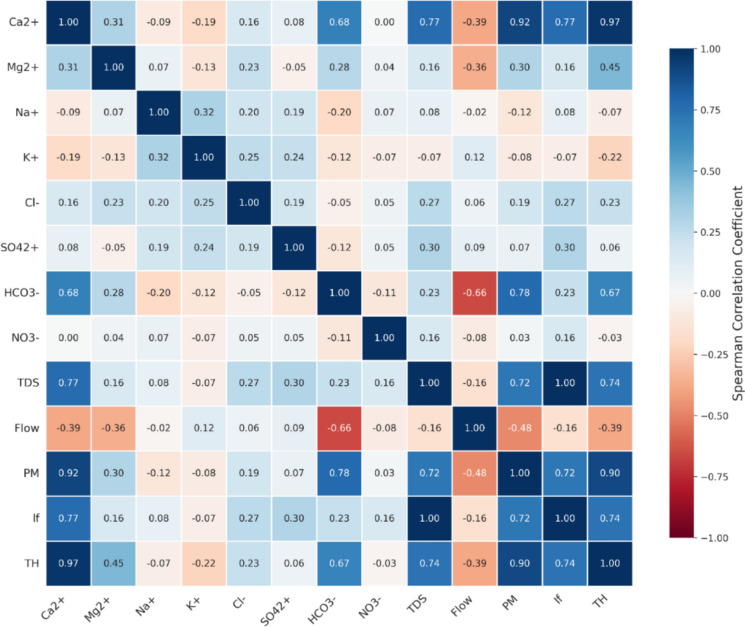
Spearman correlation matrix between the main physicochemical parameters of groundwater

The analysis of the correlations (Figs. 9 and 10) between the parameters mentioned above revealed a very strong correlation between total hardness (TH) and Ca^2+^ (Pearson and Spearman coefficients: 0.97). Similarly, a high correlation was observed between mineralization potential (MP) and Ca^2+^ (Pearson: 0.80; Spearman: 0.92). In addition, strong mineralization is also noted between the mineralization potential (MP) and HCO_3_^−^ (Pearson: 0.71; Spearman: 0.78). Moderate correlations appear between Ca^2+^ and HCO_3_^−^ (Pearson: 0.72; Spearman: 0.68), as well as between total dissolved solids (TDS) and total hardness (Pearson: 0.76; Spearman: 0.74). In contrast, NO_3_^−^, Na^+^, K^+^, Cl^−^ and SO_4_^2−^ ions show weak or negative correlations with most other parameters.

Also, these correlations allowed us to detect, on the one hand significant differences between the Pearson and Spearman correlations for the associated variables (Table 4), bicarbonate and flow, flow and mineralization potential, Calcium and flow, Potassium and Chlorides and on the other hand negative correlations and especially Spearman correlations between flow and mineralization, flow and calcium, flow and bicarbonates.

The comparison between the Pearson and Spearman correlation reveals notable differences in the relationships between several pairs of groundwater parameters. For example, the correlation between Bicarbonate and Flow Rate is 0.161 for Pearson but -0.657 for Spearman, a difference of 0.818. Similarly, the relationship between Flow Rate and MP shows a Pearson value of 0.079, which contrasts with −0.485 in Spearman, yielding a difference of 0.564. The Pearson correlation between Calcium and Flow Rate is 0.150, while the Spearman correlation is −0.390, a difference of 0.540. Finally, the correlation between Potassium and Chloride is 0.613 for Pearson and 0.252 for Spearman, a difference of 0.361. These disparities suggest that while Pearson assesses linear relationships, Spearman may be more sensitive to non-linear associations or outliers, highlighting the importance of selecting the appropriate method based on data characteristics.

#### Modelling of HCO_3_^−^ and flow relationships

The two numerical variables were identified, where the flow rate Q is an explanatory variable for the concentration of HCO₃⁻in the model. Based on the 71 available observations, a third-degree polynomial regression was fitted.

**Fig. 11:**
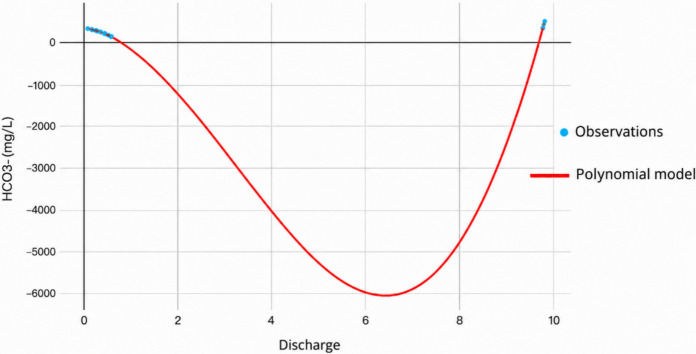
Cubic model describing the nonlinear relationship between discharge and bicarbonate concentrations

The generated graph is a scatter plot (HCO₃⁻ observed) with the cubic model curve f(Q) superimposed, showing the nonlinear trend between flow and HCO₃⁻ concentration (Fig. 11).

The trend graph f(Q) = HCO₃⁻ shows a clearly non-linear relationship between flow rate and bicarbonate concentration. For low flows (Q < 1 L/s), the curve shows high HCO₃⁻ values, reflecting longer residence times and more advanced water mineralization. As the flow increases, the trend is towards a decrease in HCO₃⁻ concentrations, consistent with dilution by newer, less mineralized waters. The polynomial curve closely follows the scatter plot in the densest region of the data, indicating that the model correctly captures the main structure of the HCO₃⁻–Q relationship and provides a physically consistent representation of the system's hydrogeochemical behavior.

Using the Python programming language, we have adapted the polynomial regression model to the dataset where the values β0, βn1, and C are determined.

Fitting of the function f(Q) = HCO_3_ to the dataset polynomial regression model showed us that the best fit for these data is a third-degree polynomial (Eq. 2):

2 $$Simulated\;HC{O}_{3}^{-}=349.99-174.44\times Q-406.94\times {Q}^{2}+43.69\times {Q}^{3}$$

The performance of the cubic polynomial model relating the HCO₃⁻ concentration to the Q flow rate was evaluated using standard statistical indicators within an "uncertainty analysis" in order to quantify the quality of the fit. In this approach, the root of the root mean squared error (RMSE), the mean absolute error (MAE), and the coefficient of determination (R^2^) provide complementary measure of the accuracy of the predictions and the share of variability explained by the model.

**Fig. 12:**
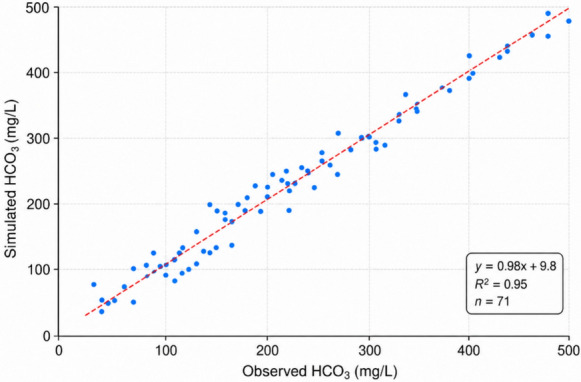
Relationship between simulated and observed HCO3^−^

The uncertainty analysis was conducted based on the 71 pairs of simulated and observed HCO₃⁻ concentrations. The individual errors (observed–simulated) show a distribution centered around zero, without marked bias. The global indicators show an absolute mean error MAE ≈ 15 mg/L and a RMSE mean square error ≈ 19 mg/L, for concentrations varying globally between 120 and 500 mg/L. Linear regression between simulated and observed values provides a coefficient of determination R^2^ ≈ 0.95, with a slope close to unity and a low y-intercept (Fig. 12), indicating a good overall fit of the model and a moderate relative uncertainty over the range of concentrations studied.»

#### Principal component analysis

**Fig. 13:**
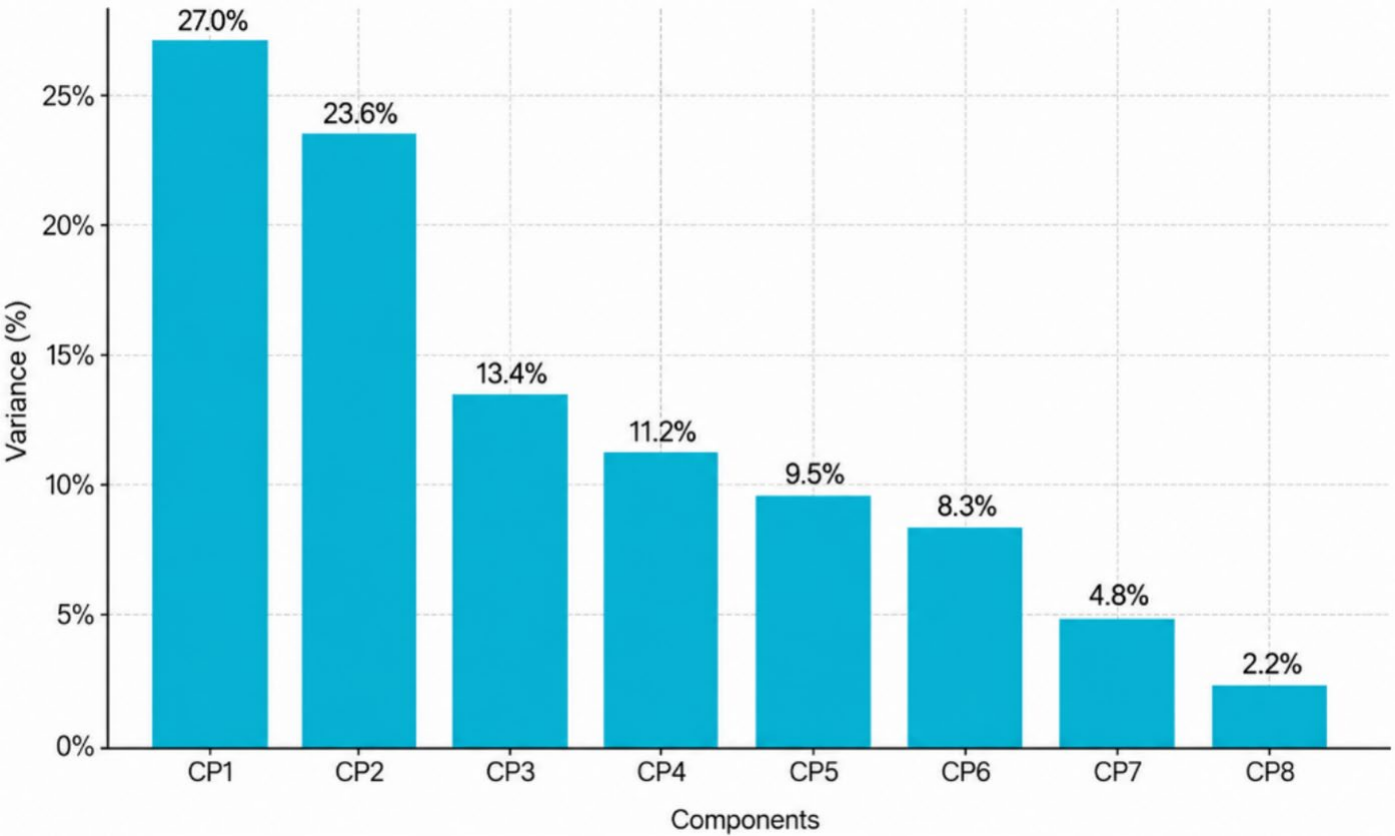
Distribution of the PCA variance for the chemical elements of the waters of the Aïn Taga spring

The principal component analysis (PCA) performed on the 71 water samples in the study area revealed eight variables, of which the first five principal components, representing 84.6% of the total variance, can satisfactorily interpret the mineralization processes in this karst network (Fig. 13).

**Fig. 14:**
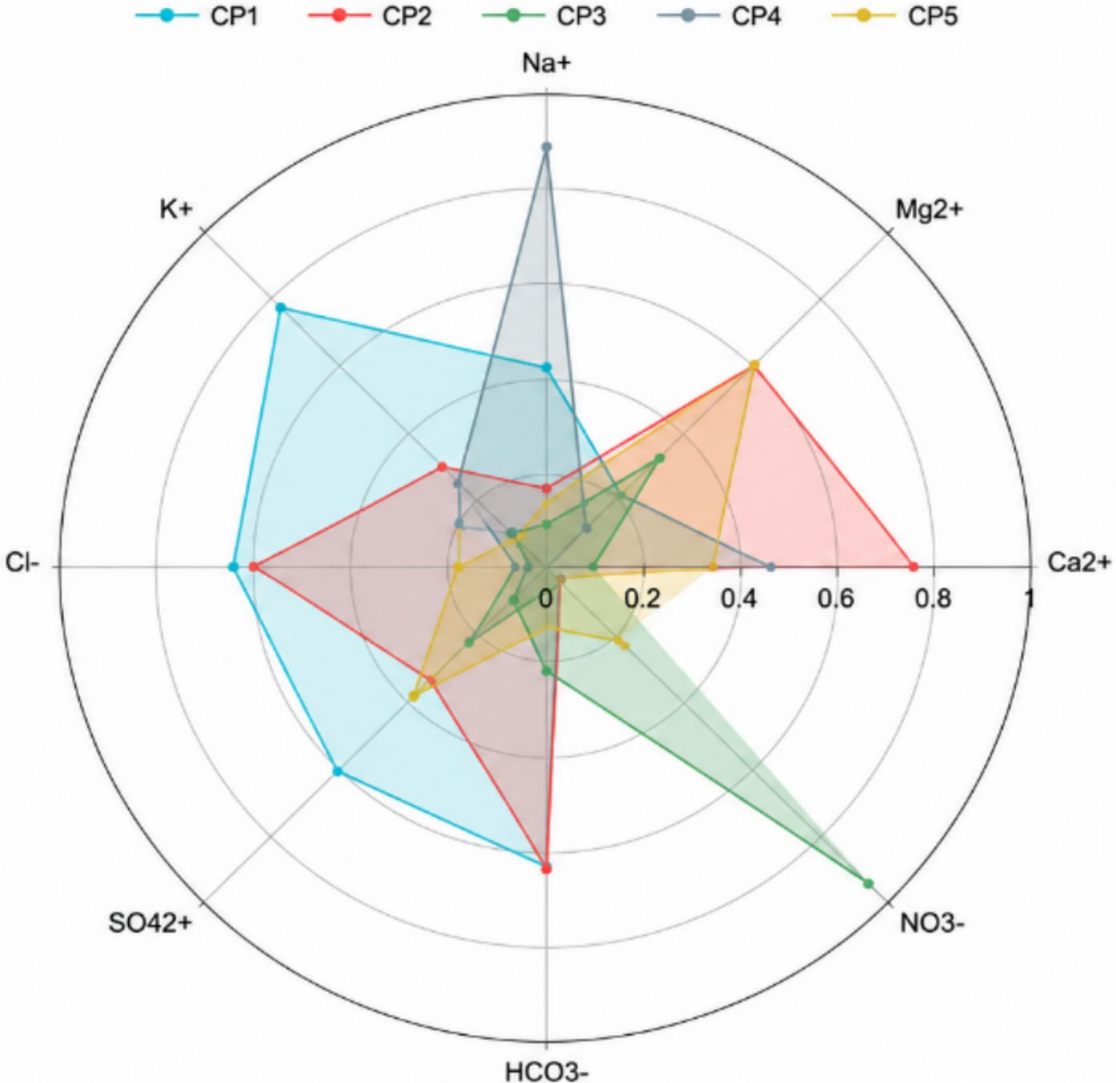
Absolute correlations of variables with the first 5 Principal Components

The mineralization factors identified (Fig. 14) are distributed as follows: the salinity factor (CP1, 27.1%) opposes mineralization of saline origin, marked by high concentrations of K⁺, Cl⁻ and SO_4_^2^⁻, reflecting evaporation or saline contamination, to carbonate mineralization characterized by HCO_3_^⁻^ contents, resulting from the interaction of water and carbonate rock; the total hardness factor (CP2, 23.6%) reflects the classic dissolution mineralization of carbonate and evaporitic rocks, with significant inputs of Ca^2^⁺, HCO₃⁻, Mg^2^⁺ and Cl⁻; the nitrate contamination factor (CP3, 13.4%), dominated by the presence of nitrates and moderately associated with magnesium, indicates pollution of anthropogenic origin (agricultural practices or sanitation) independent of natural processes; the specific sodium factor (CP4, 11.2%) is centred on sodium, as opposed to potassium and sulphates, reflecting mineralisation linked to the alteration of sodium silicates or ion exchanges; finally, the magnesium-sulfate factor (CP5, 9.3%) reveals an opposition between magnesium and sulfates, illustrating the differential dissolution of magnesium minerals as well as the processes of precipitation or dissolution of sulfates.

### Water quality classification

Water quality classification is an essential assessment tool for characterising water resources according to standardised physico-chemical criteria. This systematic approach aims to evaluate the suitability of water for different uses (human consumption, irrigation, industrial activities) and to establish quality thresholds based on rigorous scientific standards.

The primary objective of this classification is to provide a clear interpretative framework that enables water resource managers, health authorities, and users to rapidly assess the quality of a water resource and its limitations on use. This assessment is based on the analysis of key parameters, such as hardness and salinity, which are fundamental indicators of water's chemical composition.

Water hardness represents the total concentration of calcium (Ca^2^⁺) and magnesium (Mg^2+^) ions, expressed in French degrees (°F). This classification is based on international standards (Table 2):

**Table 2:** Classification of water hardness according to international standards

Category	Hardness beach	Characteristics
Very Gentle	0–8°F (0–80 mg/L CaCO3)	Aggressive water, corrosive to the pipes
Gentle	8–15°F (80–150 mg/L CaCO3)	Acceptable quality, low-scale formation
Moderately hard	15–25°F (150–250 mg/L CaCO3)	Optimal quality for consumption
Hard	25–35°F (250–350 mg/L CaCO3)	Scale formation, heavy use of detergents
Very hard	> 35°F (> 350 mg/L CaCO3)	Significant scaling problems

Salinity evaluates the total concentration of dissolved salts, usually expressed as electrical conductivity (μS/cm) or TDS (Total Dissolved Solids). The classification thresholds are shown in Table 3

**Table 3:** Classification of water hardness according to international standards

Category	TDS	Quality
Freshwater	< 500 mg/L	Excellent quality
Lightly salted	500–1000 mg/L	Good quality
Moderately salty	1000–2000 mg/L	Acceptable quality
Highly salty	> 2000 mg/L	Limited Use

The analysis of the 71 samples reveals a characteristic distribution according to standard classification criteria (Table 4). This distribution indicates a hydrogeological context dominated by carbonate aquifers. The predominance of hard to very hard water reflects the geological influence of limestone and dolomite formations, typical of sedimentary aquifer systems.

**Table 4:** Classification result of Aïn Taga spring water hardness according to international standards

Criterion	Category	Number (%)
Hardness	Hard	41 (57.7%)
	Moderately hard	16 (22.5%)
	Very hard	14 (19.7%)
Salinity	Lightly salted	66 (93.0%)
	Moderately salty	5 (7.0%)

### Hydrogeochemical analysis

The analysis of saturation indices for each sample is an essential step in understanding the hydrogeochemical evolution of the karst waters of Ghar Boumaâza in the Tlemcen region (Algeria). The saturation indices (SI) for calcite, dolomite, and are used to evaluate the state of equilibrium or imbalance of the waters with respect to these minerals, and thus to identify the dominant dissolution or precipitation processes within the aquifer. The saturation indices calculated from the major chemical analyses are related to total dissolved solids (TDS). The interpretation of the statistical distribution of the SI and its evolution as a function of the TDS highlights the system's geochemical dynamics, the preponderant role of water-carbonate rock interactions, and the absence of a significant influence from evaporites. These data offer a precise diagnosis of the hydrogeochemical functioning of the Ghar Boumaâza karst and the quality of the aquifer reservoir.

#### Saturation indices per sample

**Fig. 15:**
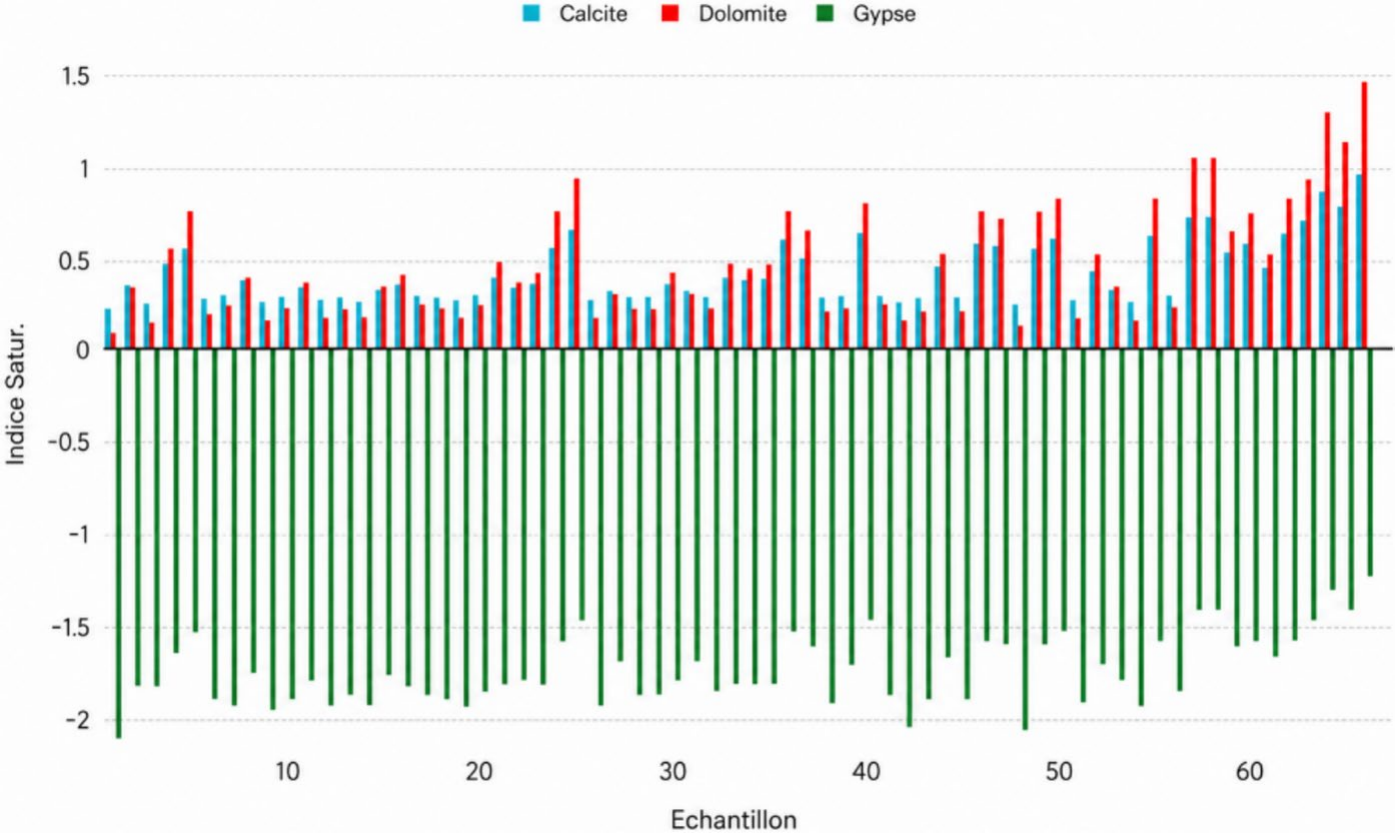
Mineral saturation indices (calcite, dolomite, gypsum) per sample

The graph (Fig. 15) reveals distinct geochemical behaviors for each mineral:

- *Calcite*: All samples show positive indices (SI > 0), indicating widespread supersaturation. This supersaturation ranges from 0.22 to 0.96, with a mean of 0.30, indicating a dynamic equilibrium between dissolution and potential precipitation during CO3 degassing.
- *Dolomite*: Also supersaturated in all samples (SI > 0), with more variable values (0.09–1.50). This strong supersaturation reflects the very slow kinetics of dolomite precipitation compared to its dissolution, a process typical of karst environments.
- *Gypsum*: Consistently undersaturated (SI < 0) with values between −2.11 and −1.23. This constant undersaturation confirms the absence of evaporites in the aquifer and the exclusively carbonate origin of the mineralization.

#### Relationship saturation indices and total dissolved salts (TDS)

**Fig. 16:**
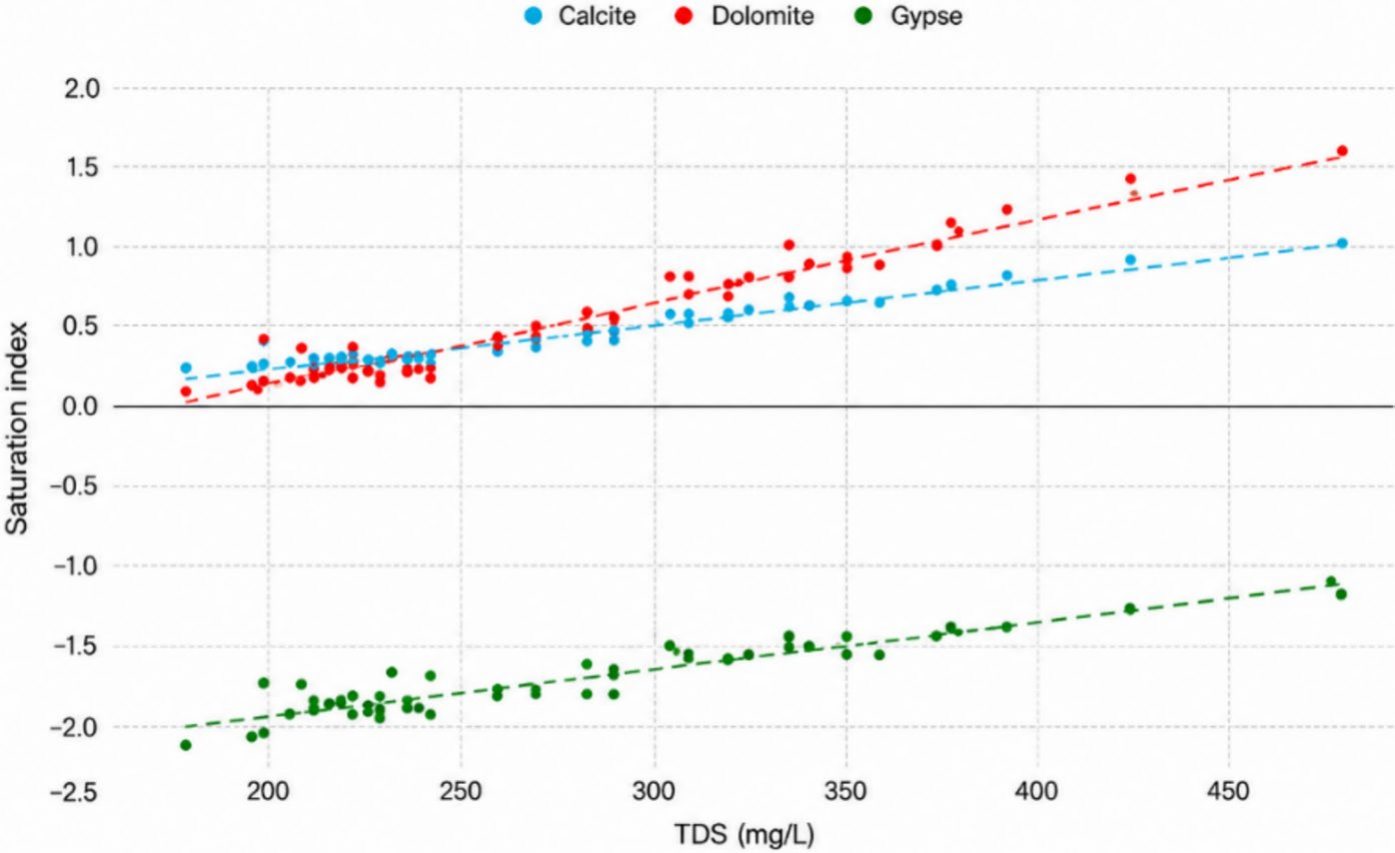
Relationship of the Saturation Index and Total Mineralization (TDS)

The correlation between saturation indices and total mineralization (Fig. 16) reveals the processes of hydrochemical evolution:

- *Calcite and Dolomite*: Strong positive correlation with TDS (R^2^ > 0.8), demonstrating that the increase in mineralization is accompanied by increasing carbonate supersaturation. This evolution reflects progressive geochemical ageing of the waters, with increasing equilibration with the carbonate phases.
- *Gypsum*: Moderate positive correlation with TDS, but systematic maintenance of undersaturation. The relative improvement of the showing with mineralization suggests limited sulphate inputs (oxidation of sulphides, dissolution of residual evaporites) but insufficient to reach equilibrium.

#### Statistical distribution of the saturation indices by mineral

**Fig. 17:**
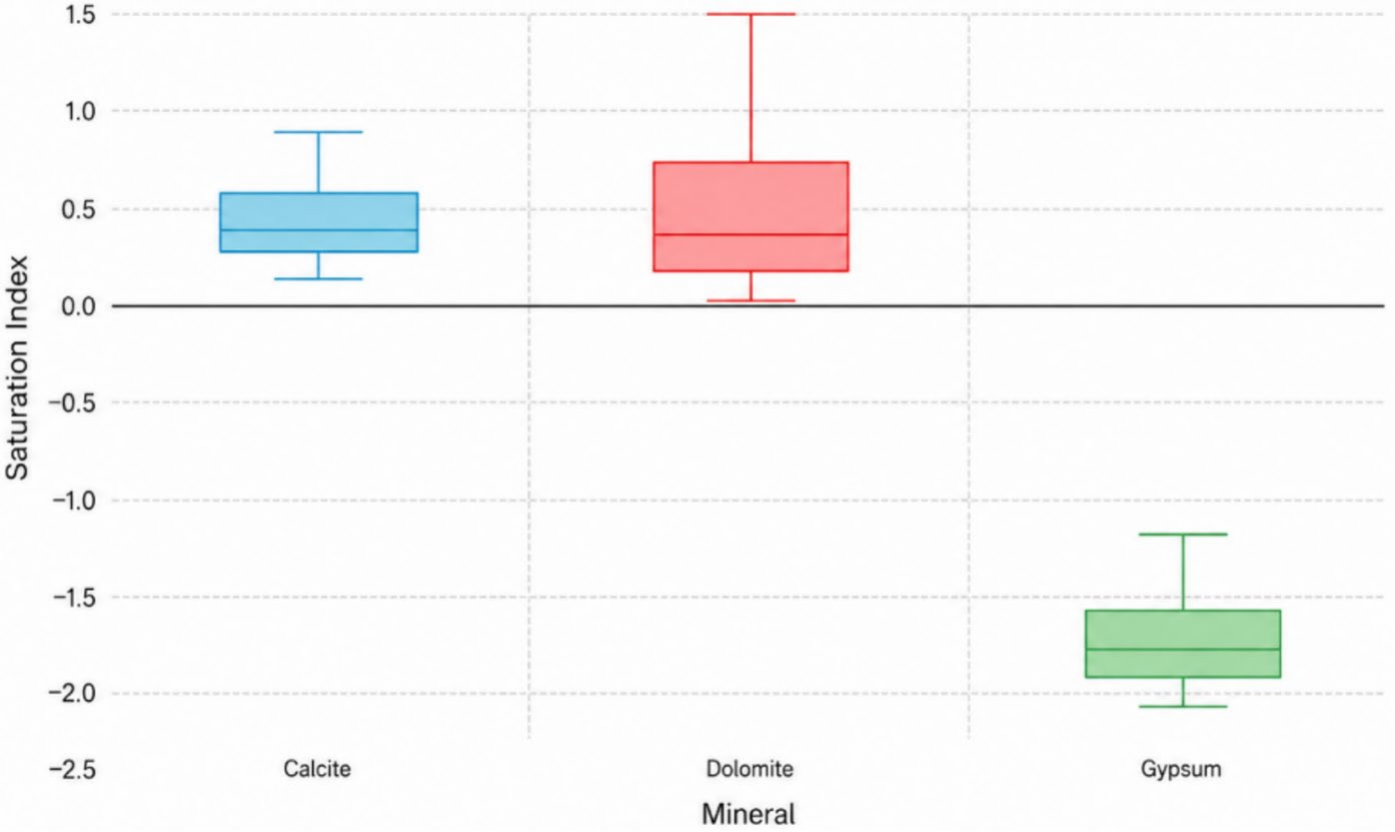
Distribution of Mineral Saturation Indices

The distribution of the showings reveals three distinct geochemical signatures (Fig. 17):

- *Calcite*: Homogeneous and positive distribution (median ≈ 0.32), with low dispersion, characteristic of a system in equilibrium with the dominant carbonate phase.
- *Dolomite*: More dispersed distribution with median at 0.41 and presence of extreme values, reflecting the heterogeneity of dolomitic dissolution and the different water–rock contact times.
- *Gypsum*: Negative distribution concentrated around −1.7 with moderate dispersion, confirming uniformity of sulphate undersaturation throughout the aquifer.

This hydrogeochemical study highlights a mature Mediterranean karst system, characterized by the major control of groundwater chemical composition by the dissolution of carbonates (calcite and dolomite). The geochemical evolution of the waters is part of a gradient of increasing mineralization, reflecting a progression towards equilibrium with the carbonate phases. The absence of evaporites in the local context limits secondary sulphate processes, while the observed spatial variability reflects the structural heterogeneity specific to karst. This geochemical profile, typical of Mediterranean carbonate aquifers, illustrates the differentiated circulation and progressive equilibration between the seepage waters and the carbonate rock matrix of the Ghar Boumaâza system.

## Discussion

### Graphical and compositional analysis

The hydrochemical signature of the Aïn Taga spring is predominantly controlled by carbonate dissolution processes. The strong prevalence of the Ca–Mg–HCO₃ facies, together with high correlations between Ca^2^⁺, Mg^2^⁺, and HCO₃⁻, confirms that water–rock interaction within limestone and dolomitic formations is the principal driver of mineralization. This behavior is typical of mature Mediterranean karst aquifers, where groundwater rapidly equilibrates with carbonate lithologies during subsurface circulation.

The results of the HCO_3_^−^ = f(Q) model highlight a clearly nonlinear relationship between HCO₃⁻ concentration and flow, satisfactorily described by a cubic polynomial that reflects both strong mineralization at low flow and progressive dilution at high flow. For low flows (Q < 1 L/s), high HCO₃⁻ concentrations suggest prolonged residence times in the karst reservoir, favouring carbonate dissolution, whereas increased flow is accompanied by an average decrease in HCO₃⁻ content, linked to the addition of newer, less mineralized water mobilized during high water. The scatter plot is correctly reproduced by the third-degree polynomial f(Q), which faithfully renders the structure of the relationship and the hydrogeochemical behavior of the source. This choice of model is supported by the marked curvature of the trend, which is difficult to represent with a linear or quadratic fit, as well as by the calibration procedure based on a set of learning (70%) and testing (30%), which limits overfitting.

The statistical performance is satisfactory, with an MAE of about 15 mg/L and an RMSE close to 19 mg/L for concentrations between 120 and 500 mg/L, an R^2^ of around 0.95, a simulated/observed regression slope close to unity, and a low y-intercept, as well as a residue distribution centered on zero, without any noticeable bias. All of these elements confirm the predictive reliability of the model and its hydrogeological interest: the combination of high concentrations at low flow and dilution at high flow is characteristic of a karst system combining a slow, highly mineralized reservoir and rapid inputs of cooler water, so that the HCO₃⁻–Q relationship constitutes an operational tool to link the hydrodynamic regime and the chemical signature, simulate the evolution of bicarbonates during flood periods and estimate concentrations from continuous flow measurements, within the limits of the Q domain and the conditions covered by the 71 observations.

Saturation index calculations further reinforce this interpretation. The systematic supersaturation with respect to calcite and dolomite indicates that groundwater approaches equilibrium with carbonate minerals during flow through the aquifer and may experience additional CO₂ degassing upon emergence at the spring. Such conditions are characteristic of karst systems where dissolution dominates in the subsurface, followed by potential secondary precipitation near discharge zones. In contrast, the persistent undersaturation with respect to gypsum indicates that evaporitic formations do not significantly contribute dissolved sulfate, suggesting that most sulfate originates from minor accessory minerals or anthropogenic sources rather than from natural evaporite dissolution.

Although natural geochemical processes dominate, several indicators reveal localized anthropogenic impacts. Elevated concentrations and high variability of NO₃⁻, Cl⁻, SO₄^2^⁻, Na⁺, and K⁺ suggest episodic external inputs linked to agricultural activities and domestic wastewater. These ions show weak correlations with carbonate-derived parameters, supporting the interpretation that they originate from surface sources rather than bedrock dissolution.

### Statistical and component analysis

Principal Component Analysis helps distinguish these influences. The first components are associated with natural carbonate mineralization, whereas separate components highlight nitrate and salinity-related variables, reflecting human-derived contamination. Importantly, these anthropogenic signals appear superimposed on a stable natural geochemical background, indicating that while water quality remains generally suitable for most uses, the karst system is vulnerable to surface-derived pollution. This vulnerability is consistent with the high permeability and rapid recharge pathways typical of conduit-dominated karst aquifers.

One of the most significant findings of this study is the strong nonlinear relationship between spring discharge and bicarbonate concentration. The marked negative Spearman correlation between flow rate and HCO₃⁻ concentration indicates a dilution effect during high-flow events. During periods of intense recharge, low-mineralized meteoric water is rapidly transmitted through conduits, diluting the more mineralized baseflow stored in the rock matrix. This dual-flow behavior, fast conduit flow superimposed on slower diffuse flow, is a defining characteristic of karst aquifers and explains the observed nonlinear concentration–discharge relationship.

The polynomial regression model captures this behavior effectively, demonstrating that hydrochemical responses cannot be adequately described using simple linear assumptions. Such nonlinear dynamics reflect the complex mixing processes and variable residence times that govern solute transport in karst systems. These results emphasize that hydrochemical monitoring in karst environments must consider hydrological variability to avoid misinterpreting short-term dilution as long-term geochemical change.

The relationship between saturation indices and total dissolved solids indicates progressive geochemical evolution along flow paths. Increasing mineralization is accompanied by greater carbonate supersaturation, reflecting longer water–rock contact times and more advanced equilibration. The relatively narrow distribution of calcite saturation indices suggests a system approaching steady geochemical equilibrium, whereas the wider dispersion in dolomite saturation indices reflects slower reaction kinetics and lithological heterogeneity.

### Water quality and hydrogeochemistry

Aïn Taga system represents a mature carbonate karst aquifer in which natural dissolution processes dominate but are periodically modified by rapid recharge events and localized anthropogenic inputs. This combination of strong natural buffering and high vulnerability is typical of Mediterranean semi-arid karst environments.

The findings highlight both the resilience and fragility of the Aïn Taga karst resource. While natural carbonate buffering generally maintains good chemical quality, the system’s rapid recharge and conduit flow pathways allow contaminants from the surface to reach the spring with minimal attenuation. Seasonal variations in discharge further amplify vulnerability, as high-flow periods promote rapid transport of pollutants.

Effective management, therefore, requires regular monitoring of nitrate, chloride, and sulfate, particularly during recharge seasons. Land-use regulation around recharge zones and improved wastewater management are essential to prevent long-term degradation of this strategic water resource.

## Conclusions

This study provides the first long-term hydrogeochemical characterization (2014–2024) of the Aïn Taga karst spring, the main outlet of the Ghar Boumaâza system in northwestern Algeria. The dominance of the Ca–Mg–HCO₃ facies, together with systematic supersaturation with respect to calcite and dolomite, shows that carbonate dissolution in limestone and dolomitic formations is the primary control on water mineralization, whereas evaporitic formations play only a minor role. The cubic HCO₃⁻–Q model reveals a clear nonlinear relationship between discharge and bicarbonate concentrations, with high mineralization at low flow (long residence times and strong water–rock interaction) and progressive dilution during high-flow periods, and it reproduces the observations with very good statistical performance. This behavior reflects the duality between a slow, highly mineralized reservoir and rapid recharge through karst conduits, confirming that the concentration–discharge relationship is an operational proxy for linking hydrodynamic regime and chemical signature at the spring. Multivariate statistical analyses distinguish a stable natural geochemical background, controlled by carbonate dissolution, from localized anthropogenic inputs associated with agricultural activities and domestic wastewater, mainly expressed by NO₃⁻, Cl⁻, SO₄^2^⁻, Na⁺, and K⁺. These results highlight both the overall good chemical quality of the water and the high vulnerability of this strategic karst resource to surface contamination. The integrated methodological framework developed here—combining classical hydrochemistry, saturation indices, nonlinear HCO₃⁻–Q modelling, and multivariate statistics—provides a transferable tool for diagnosing processes, tracking weathering dynamics, and supporting monitoring and protection strategies in vulnerable karst aquifers under increasing climatic and anthropogenic pressures.

## Data Availability

The datasets used and/or analyzed during the current study are available from the corresponding author upon reasonable request.
